# Does early letermovir initiation delay engraftment?

**DOI:** 10.1128/aac.00371-26

**Published:** 2026-06-04

**Authors:** Léna Royston, Catia Vieira Gomes, Ming Zou, Christian Van Delden, Laurent Kaiser, Yves Chalandon, Stavroula Masouridi-Levrat, Dionysios Neofytos

**Affiliations:** 1 Division of Infectious Diseases, University Hospital of Geneva 27230, Geneva, Switzerland; 2 University Hospital Basel 30262, Basel, Switzerland; 3 Division of Hematology, Bone Marrow Transplant Unit, University Hospital of Geneva, Geneva, Switzerland; Chinese Academy of Medical Sciences & Peking Union Medical College, Beijing, China

**Keywords:** side effect, antiviral, engraftment, hematopoietic cell transplantation, cmv, cytomegalovirus, letermovir

## Abstract

Although letermovir prophylaxis has drastically reduced cytomegalovirus (CMV) infections in allogeneic hematopoietic cell transplantation recipients (allo-HCTR), the broader impact of letermovir prophylaxis remains unclear. We retrospectively analyzed hematological outcomes in CMV-seropositive allo-HCTR (R+) from the pre- and letermovir periods. Among 314 consecutive allo-HCTR+, engraftment was significantly delayed in allo-HCTR+ in the letermovir period, even after removing potential confounders, and despite improved survival. Similar trends were found after reviewing the literature, and this observation should raise vigilance.

## INTRODUCTION

Letermovir prophylaxis post-allogeneic hematopoietic cell transplantation (allo-HCT) allowed a great decline in the incidence of clinically significant CMV infections (csCMVi) and related morbidities (1). More broadly, real-life cohort studies and meta-analyses have highlighted the beneficial influence on other post-HCT outcomes, including CMV treatment-related toxicities, graft-versus-host disease (GvHD) incidence, healthcare utilization and costs, and mortality (2, 3). Nevertheless, the larger impact of letermovir universal prophylaxis remains unclear, and there might be counterparts of inhibiting CMV low-grade replication, such as the delay in CMV-specific immune reconstitution (4), or even of the drug itself, despite its apparently well-tolerated profile. In a previous study, we observed a prolonged time-to-engraftment in patients receiving letermovir (*n* = 17, 21.2 days) compared to matched controls (*n* = 34, 17.4 days; *P* = 0.004) (5). To further investigate this observation, we retrospectively analyzed time-to-engraftment and other hematological outcomes in a larger allo-HCT recipient cohort in the pre-letermovir and letermovir study periods.

We conducted a single-center cohort study of consecutive adult allo-HCT CMV-seropositive recipients (R+) who received their first HCT at our institution from 16 November 2015 to 31 December 2023. The study was approved by the local ethics committee (protocols 2020-00059/2020-02120/2025-01263), and patients signed an informed consent form for data utilization for clinical research as part of an existing protocol (14-220/2025-01263). The primary objective was to describe time-to-engraftment of allo-HCTR+ in the pre- and letermovir periods. Secondary objectives included the description of other non-virological clinical outcomes (all-cause mortality, grade ≥2 acute GvHD, hematological relapse by 1-year posttransplant) in the pre- and letermovir study periods. Recipient CMV-serostatus of allo-HCT recipients is defined during pre-HCT transplant infectious disease consultation based on our institutional algorithm (6, 7). Engraftment is defined by neutrophil engraftment, which corresponds to the first of three consecutive days with an absolute neutrophil count of ≥0.5 × 10^9^/L in peripheral blood. Letermovir primary CMV prophylaxis was introduced on 1 May 2019, so the study period was divided into pre- and letermovir periods, before and after 1 May 2019, respectively. Letermovir was administered from posttransplant day (d) 1 to d100 until 31 December 2020 to all CMV donor (D)−/R + patients and to all allo-HCT R+ starting 1 January 2021 (5, 8). In addition, allo-HCTR+ with early grade ≥2 acute GvHD received primary letermovir prophylaxis until tapering to <10 mg/d of prednisone. Preemptive treatment for csCMVi was initiated for CMV-DNAemia ≥150 IU/mL until 31 December 2022, and thereafter at ≥500 or ≥150 IU/mL in patients receiving or not receiving letermovir prophylaxis, respectively. Kaplan-Meier analyses of time-to-engraftment, all-cause mortality, hematological relapse, and acute GvHD ≥grade 2 were performed for the first year post-HCT. Log-rank and Peto-Peto tests were used to assess differences in cumulative incidences and Kaplan-Meier analyses, as appropriate. Multivariable Cox proportional hazards regression was used to estimate the association between patients' and HCT characteristics with the time-to-engraftment outcome.

**Fig 1:**
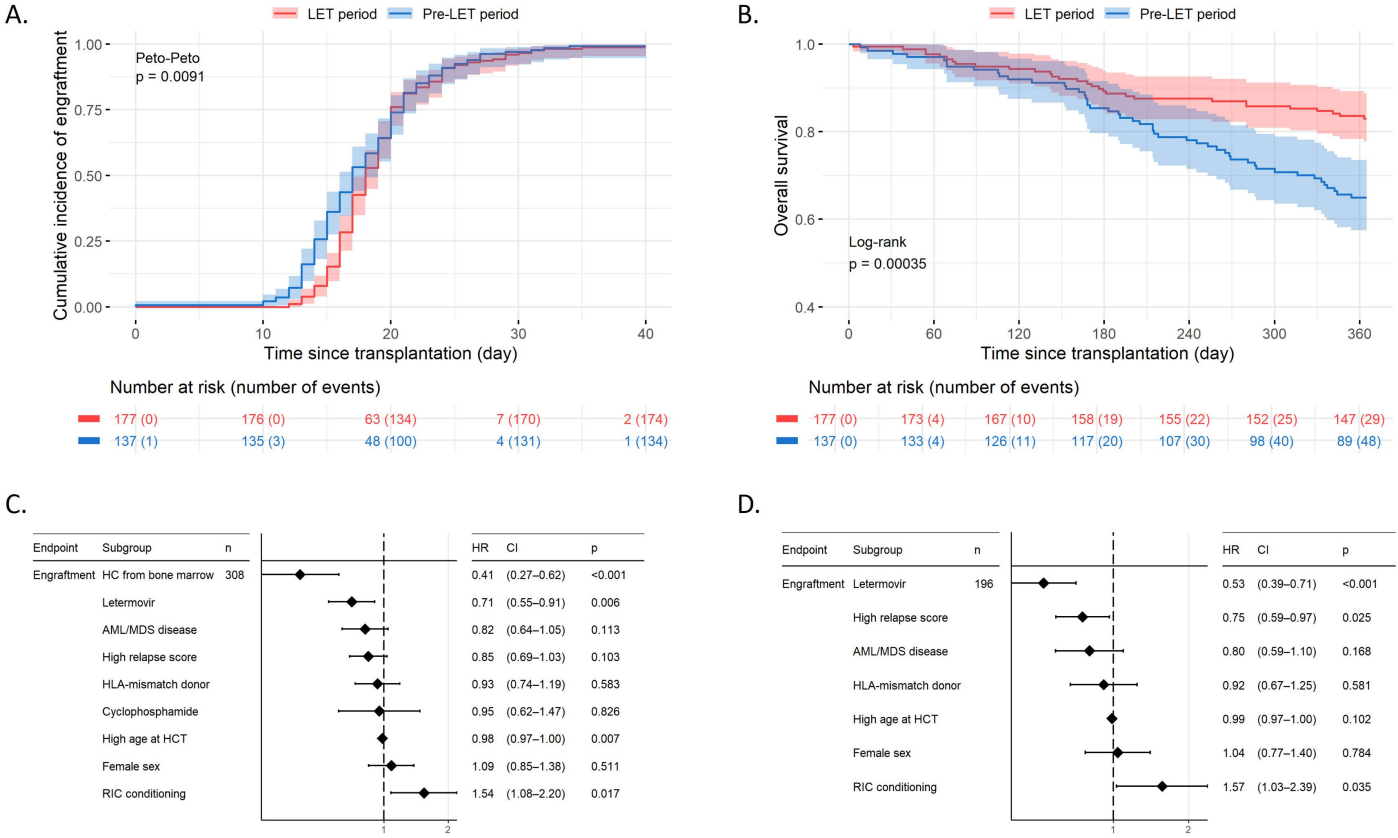
Time-to-engraftment and all-cause mortality in the pre-letermovir and letermovir study periods. (**A and B**) Cumulative incidence of engraftment (**A**) and all-cause mortality (**B**) in the pre-letermovir (blue) or letermovir (red) study periods. (**C and D**) Risk factors for delayed engraftment using multivariable Cox regression analysis in the whole population (**C**) and only in allogeneic hematopoietic cell transplant recipients with peripheral blood stem cells as stem cell sources and who did not receive cyclophosphamide as GvHD prophylaxis (**D**). Hazard ratios below 1 correspond to a delay in the time-to-engraftment, whereas hazard ratios above 1 correspond to shorter time-to-engraftment. LET: letermovir; HR: hazard ratio; CI: confidence interval.

Overall, 137 (65%) and 177 (60%) allo-HCT R+ were considered as CMV R+ during the pre- and letermovir periods, respectively. Patient characteristics were similar between the two periods, except for higher rates of peripheral blood stem cells (PBSC) versus bone marrow (BM) stem cells as HCT source (94.4% versus 81.8%, *P* < 0.001) and of posttransplant cyclophosphamide-based regimens for GvHD prophylaxis (PTCy) (37.9%, versus 29.5%, *P* < 0.001) in the letermovir study period (Table 1). Engraftment was significantly delayed in the letermovir period, with a median time to engraftment of 18 versus 17 days post-HCT in the pre-letermovir period (*P* = 0.009, Fig. 1A). One-year all-cause mortality was significantly decreased in the letermovir period (*P* < 0.001, Fig. 1B), whereas 1-year cumulative incidence of acute GvHD and hematologic relapse were similar between the study periods (*P* = 0.7 and *P* = 0.84, respectively). Relevant patient and transplant-related characteristics were evaluated as potential predictors of earlier engraftment using multivariable Cox regression analysis (Fig. 1C). Letermovir prophylaxis (hazard ratio [HR] 0.71, 95% confidence interval [CI] 0.55–0.91, *P* = 0.006). Bone marrow (BM) source (HR 0.41, 95% CI 0.27–0.62, *P* < 0.001) and higher age at HCT (HR 0.98, 95% CI 0.97–0.99, *P* = 0.008) were associated with slower engraftment time, while reduced-intensity conditioning (RIC) regimens were associated with shorter time-to-engraftment (HR 1.54, 95% CI 1.08–2.20, *P* = 0.017). No significant associations were found between those independent variables. Considering the important impact of BM source on engraftment, we performed the same analyses after excluding allo-HCTR with BM source. Letermovir was still associated with slower engraftment in patients receiving HCT from PBSC (Fig. S1). Due to the different proportions of PTCy administration between the two periods and its potential effect on engraftment, another multivariable Cox regression analysis was performed only in patients who received a transplant from PBSC and who did not receive PTCy (Fig. 1D). In those patients, administration of letermovir remained significantly associated with slower engraftment time (HR 0.53, 95% CI 0.39–0.71, *P* < 0.001), and the difference in time-to-engraftment between the letermovir (median 18 days) and pre-letermovir (median 16 days) periods was even larger (*P* < 0.001).

**TABLE 1:** Population characteristics in the pre-letermovir and letermovir periods^*a*^^,^^*b*^

HCT procedures in CMVR+	Pre-letermovir period	Letermovir period	*P*-value
	*N*: 137 (%)	*N*: 177 (%)	
Age at transplant			
Median (IQR)	56 (49, 62)	56 (45, 65)	1.00
Sex			0.83
Female	52 (38.0)	64 (36.2)	
Hematologic disease			0.74
AML/MDS	84 (61.3)	106 (59.9)	
Other	53 (38.7)	71 (40.1)	
Conditioning regimen			0.67
Myeloablative	28 (20.4)	38 (21.5)	
Reduced-intensity	109 (79.6)	139 (78.5)	
Donor type			0.15
HLA-matched related	36 (26.3)	35 (19.8)	
HLA-matched unrelated	64 (46.7)	74 (41.8)	
Haplo-identical	31 (22.6)	55 (31.1)	
HLA-mismatched unrelated	6 (4.4)	13 (7.3)	
Stem cell source			<0.001
BM	25 (18.2)	10 (5.6)	
PBSC	112 (81.8)	167 (94.4)	
GvHD prophylaxis			<0.001
Cyclophosphamide-based	39 (29.5)	67 (37.9)	
Methotrexate-based	52 (38.0)	94 (53.1)	
Other	46 (33.6)	16 (9.0)	
Risk of relapse			0.22
Low	6 (4.4)	10 (5.6)	
Intermediate	89 (65.0)	125 (70.6)	
High	36 (26.3)	35 (19.8)	
Very high	6 (4.4)	7 (4.0)	

^*a*^ HCT, hematopoietic cell transplantation; CMVR+, CMV− seropositive recipient; AML, acute myeloid leukemia; SMD, myelodysplastic syndrome; BM, bone marrow; PBSC, peripheral blood stem cells; GvHD, graft-versus-host disease.

^*b*^ N, number; AML/MDS, acute myelogenous leukemia/myelodysplastic syndrome; BM, bone marrow; PBSC, periphral blood stem cells; GvHD, graft versus host disease.

In this single-center, 8-year cohort, engraftment is slightly but significantly delayed in allo-HCTR+ in the letermovir period compared to pre-letermovir, despite the reduction in post-HCT CMV reactivation incidence and improved survival (5). In line with our observation, after reviewing all published clinical trials and cohort studies (until February 2025, *n* = 40, Table S1) on letermovir prophylaxis in allo-HCTR, we identified a trend (*P* ≤ 0.1) for delayed engraftment in five out of nine studies reporting engraftment time, including the pivotal clinical trial by Marty et al. (*P* = 0.104) (1). Whether there is an underlying pathophysiology due to, for instance, an inherent drug toxicity or related to the direct or indirect effects of letermovir on viral replication and inflammation, remains to be further investigated. In the reviewed studies, time of letermovir initiation was variable, including early posttransplant, but usually during the first 7–10 days posttransplant. Considering that letermovir prophylaxis is routinely initiated on day 1 posttransplant at our center, we hypothesized that this early and longer exposure could have a potential impact on engraftment. Hence, we have changed the timing of letermovir introduction in our institution to day 7 posttransplant starting 1 June 2025. Considering the span of the study period, changes in clinical practices over time, and imbalance between groups could have impacted engraftment. For instance, PBSC transplants known to be associated with significantly faster engraftment times were more frequently performed in the letermovir study period (9). Posttransplant cyclophosphamide-based GvHD prophylaxis regimens were also more frequently used in the letermovir period and have been associated with delays in engraftment in some studies (10–12). To exclude potential confounding, we performed the same analyses after removing patients who received a graft from a BM source and/or PTCy, and the association between letermovir and slower engraftment was still present, and the difference in time-to-engraftment was even larger. It is still possible that, due to the retrospective nature of the study, unnoticed changes in population and clinical practices might have impacted time-to-engraftment. In addition, platelet engraftment and overall graft function were not investigated, and the clinical consequences of this slight difference in severe neutropenia time remain uncertain. Nevertheless, and despite these limitations, our data represent the first, to our knowledge, clinical observation of an as-yet unidentified effect of letermovir on an important clinical variable that should raise vigilance and be further investigated in the future.

## SUPPLEMENTAL MATERIAL

Supplemental material aac.00371-26-s0001.docx Figure S1 and Table S1.

ASM does not own the copyrights to Supplemental Material that may be linked to, or accessed through, an article. The authors have granted ASM a non-exclusive, world-wide license to publish the Supplemental Material files. Please contact the corresponding author directly for reuse.
