# Supplementary material for: Does early letermovir initiation delay engraftment?
Source: Antimicrob Agents Chemother. 2026 Jun 4;70(7):e00371-26. doi: 10.1128/aac.00371-26 (PMC13321813; doi:10.1128/aac.00371-26)
Supplement: Supplemental material — Figure S1 and Table S1. [file aac.00371-26-s0001.docx]

**Supplementary Figure 1.** Risk factors for delayed engraftment using multivariable cox regression analysis in allogeneic hematopoietic cell transplant recipients with peripheral blood stem cells as stem cell sources. Hazard ratios below 1 correspond to a delay in the time-to-engraftment, whereas hazard ratios above 1 correspond to shorter time-to-engraftment. LET: letermovir, HR: hazard ratio, CI: confidence interval.

**
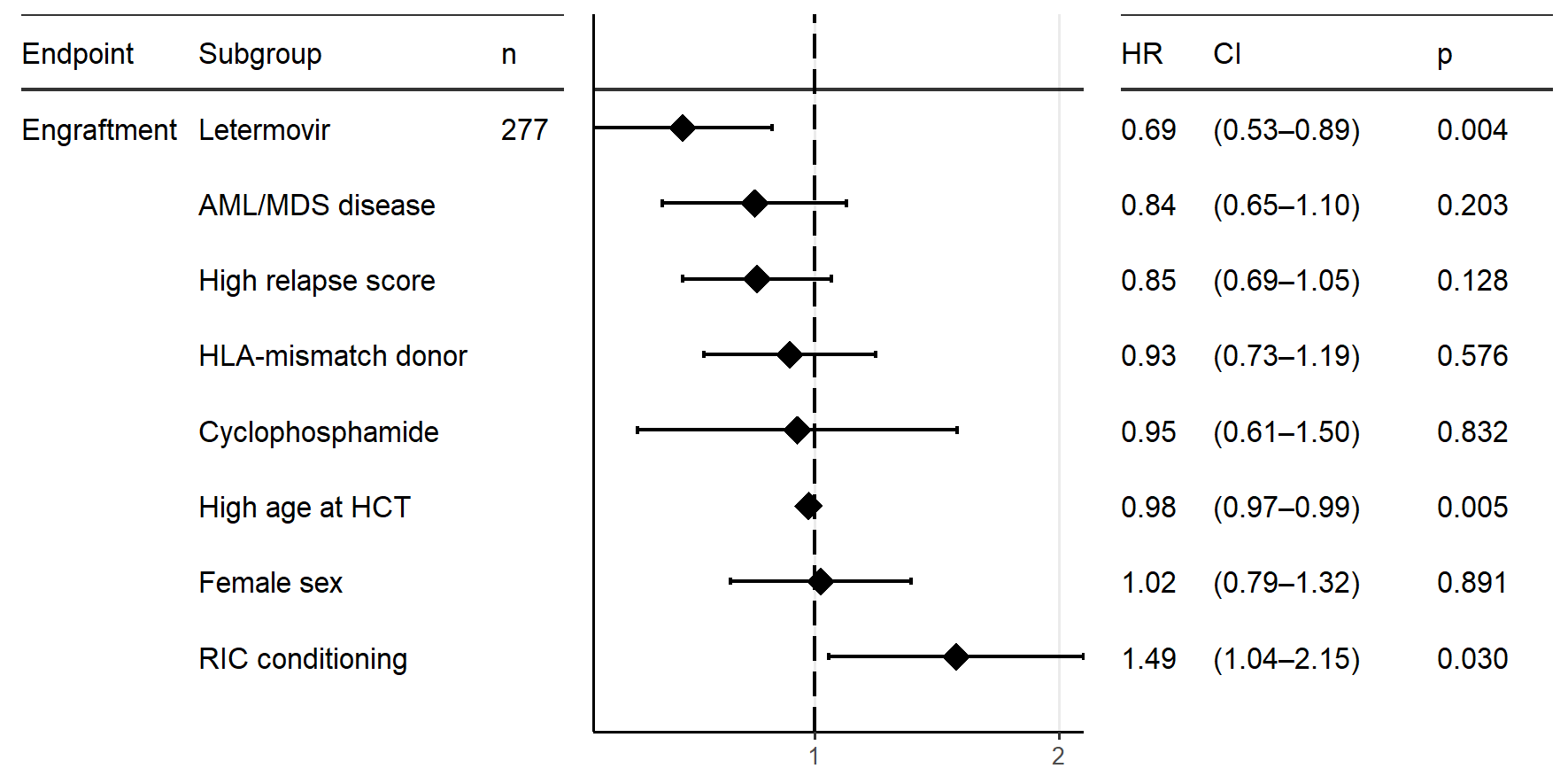
**

**Supplementary Table 1.** All published clinical trials and cohort studies (until February 2025, n=40) reporting on clinical outcomes of allogeneic hematopoietic cell transplant recipients receiving letermovir prophylaxis. N: number of patients included in the study. HCT: hematopoietic cell transplant. D: days post-HCT. Prosp: prospective study design. Retro: retrospective study design. LET: letermovir. NR: not reported.

| Studies | Study design | N | Time to engraftment | Administration post-HCT |
| --- | --- | --- | --- | --- |
| Marty, 2017^1^ | Prosp. | 373 | **Trend for delayed on LET** (*p*=0.1) | Median D9 |
| Lin, 2019^2^ | Retro. | 53 | NR |  |
| Malagola, 2020^3^ | Retro. | 60 | NR |  |
| Anderson, 2020^4^ | Retro. | 25 | **Trend for delayed on LET** (*p*=0.1, 16 vs 11) | Median D10 |
| Johnsrud, 2020^5^ | Retro. | 114 | NR |  |
| Sharma, 2020^6^ | Retro. | 32 | Similar |  |
| Łojko, 2022^7^ | Retro. | 53 | NR |  |
| Robin, 2020^8^ | Prosp. | 80 | NR |  |
| Studer, 2020^9^ | Retro. | 42 | NR |  |
| Chen, 2021^10^ | Retro. | 60 | NR |  |
| Mori, 2021^11^ | Retro. | 114 | **Trend for delayed on LET** (*p*=0.19, 19 vs 17) | Median D0 |
| Royston, 2021^12^ | Retro. | 17 | **Delayed on LET** (*p*=0.004, 21 vs 17) | Median D0 |
| Wolfe, 2021^13^ | Retro. | 119 | NR |  |
| Martino, 2021^14^ | Retro. | 204 | NR |  |
| Derigs, 2021^15^ | Retro. | 80 | NR |  |
| Cassaniti, 2021^16^ | Retro. | 75 | NR |  |
| Serio, 2021^17^ | Retro. | 13 | NR |  |
| Sassine, 2021^18^ | Retro. | 123 | Similar (*p*=0.34, 15-15) | D5 |
| Hiraishi, 2021^19^ | Retro. | 460 | NR |  |
| Beauvais, 2022^20^ | Retro. | 96 | NR |  |
| Politikos, 2022^21^ | Retro. | 28 | NR |  |
| Gabanti, 2022^22^ | Retro. | 30 | Similar (*p*=1, 17-16) | NR |
| Daukshus, 2022^23^ | Retro. | 10 | NR |  |
| Richert-Przygonska, 2022^24^ | Retro. | 13 | NR |  |
| Cheng, 2022^25^ | Retro. | 4 | NR |  |
| Freyer, 2022^26^ | Retro. | 19 | NR |  |
| Yoshimura, 2022^27^ | Retro. | 38 | NR |  |
| Mizuno, 2022^28^ | Retro. | 43 | Similar (17-18) | NR |
| Sourisseau, 2023 ^29^ | Retro. | 316 | NR |  |
| Gimenez, 2023^30^ | Prosp. | 25 | NR |  |
| Dwabe, 2023^31^ | Retro. | 116 | NR |  |
| Zavaglio, 2023 ^32^ | Prosp. | 66 | NR |  |
| Wlodarczyk, 2024^33^ | Retro. | 93 | NR |  |
| Febres-Aldana, 2024^34^ | Retro. | 383 | **Trend for delayed on LET** (*p*=0.15, 17 vs 14) | D5 |
| McGuirk, 2024^35^ | Retro. | 452 | NR |  |
| Jacobs, 2024^36^ | Prosp. | 28 | NR |  |
| Mendoza, 2024^37^ | Retro. | 166 | NR |  |
| Tan, 2024^38^ | Retro. | 176 | NR |  |
| Russo, 2024^39^ | Prosp. | 220 | NR |  |
| Han, 2025^40^ | Prosp. | 36 | NR |  |

**References**

1. Marty FM, Ljungman P, Chemaly RF, et al. Letermovir Prophylaxis for Cytomegalovirus in Hematopoietic-Cell Transplantation. *The New England journal of medicine.* Dec 21 2017;377(25):2433-2444.

2. Lin A, Maloy M, Su Y, et al. Letermovir for primary and secondary cytomegalovirus prevention in allogeneic hematopoietic cell transplant recipients: Real-world experience. *Transplant infectious disease : an official journal of the Transplantation Society.* Dec 2019;21(6):e13187.

3. Malagola M, Pollara C, Polverelli N, et al. Advances in CMV Management: A Single Center Real-Life Experience. *Frontiers in cell and developmental biology.* 2020;8:534268.

4. Anderson A, Raja M, Vazquez N, Morris M, Komanduri K, Camargo J. Clinical "real-world" experience with letermovir for prevention of cytomegalovirus infection in allogeneic hematopoietic cell transplant recipients. *Clinical transplantation.* Jul 2020;34(7):e13866.

5. Johnsrud JJ, Nguyen IT, Domingo W, Narasimhan B, Efron B, Brown JW. Letermovir Prophylaxis Decreases Burden of Cytomegalovirus (CMV) in Patients at High Risk for CMV Disease Following Hematopoietic Cell Transplant. *Biology of blood and marrow transplantation : journal of the American Society for Blood and Marrow Transplantation.* Oct 2020;26(10):1963-1970.

6. Sharma P, Gakhar N, MacDonald J, et al. Letermovir prophylaxis through day 100 post transplant is safe and effective compared with alternative CMV prophylaxis strategies following adult cord blood and haploidentical cord blood transplantation. *Bone marrow transplantation.* Apr 2020;55(4):780-786.

7. Łojko A, Styczyński J, Nasiłowska-Adamska B, et al. Real-life experiences of letermovir prophylaxis for cytomegalovirus infection in patients after hematopoietic stem cell transplantation: Polish Acute Leukemia Group (PALG) analysis. *Acta Haematologica Polonica.* 2022;53(5):350-354.

8. Robin C, Thiebaut A, Alain S, et al. Letermovir for Secondary Prophylaxis of Cytomegalovirus Infection and Disease after Allogeneic Hematopoietic Cell Transplantation: Results from the French Compassionate Program. *Biology of blood and marrow transplantation : journal of the American Society for Blood and Marrow Transplantation.* May 2020;26(5):978-984.

9. Studer U, Khanna N, Leuzinger K, et al. Incidence of CMV Replication and the Role of Letermovir Primary/Secondary Prophylaxis in the Early Phase After Allogeneic Hematopoietic Stem Cell Transplantation - A Single Centre Study. *Anticancer research.* Oct 2020;40(10):5909-5917.

10. Chen K, Arbona-Haddad E, Cheng MP, et al. Cytomegalovirus events in high-risk allogeneic hematopoietic-cell transplantation patients who received letermovir prophylaxis. *Transplant infectious disease : an official journal of the Transplantation Society.* Aug 2021;23(4):e13619.

11. Mori Y, Jinnouchi F, Takenaka K, et al. Efficacy of prophylactic letermovir for cytomegalovirus reactivation in hematopoietic cell transplantation: a multicenter real-world data. *Bone marrow transplantation.* Apr 2021;56(4):853-862.

12. Royston L, Royston E, Masouridi-Levrat S, et al. Letermovir Primary Prophylaxis in High-Risk Hematopoietic Cell Transplant Recipients: A Matched Cohort Study. *Vaccines.* Apr 12 2021;9(4).

13. Wolfe D, Zhao Q, Siegel E, et al. Letermovir Prophylaxis and Cytomegalovirus Reactivation in Adult Hematopoietic Cell Transplant Recipients with and without Acute Graft Versus Host Disease. *Cancers.* Nov 8 2021;13(21).

14. Martino M, Pitino A, Gori M, et al. Letermovir Prophylaxis for Cytomegalovirus Infection in Allogeneic Stem Cell Transplantation: A Real-World Experience. *Frontiers in oncology.* 2021;11:740079.

15. Derigs P, Radujkovic A, Schubert ML, et al. Letermovir prophylaxis is effective in preventing cytomegalovirus reactivation after allogeneic hematopoietic cell transplantation: single-center real-world data. *Annals of hematology.* Aug 2021;100(8):2087-2093.

16. Cassaniti I, Colombo AA, Bernasconi P, et al. Positive HCMV DNAemia in stem cell recipients undergoing letermovir prophylaxis is expression of abortive infection. *American journal of transplantation : official journal of the American Society of Transplantation and the American Society of Transplant Surgeons.* Apr 2021;21(4):1622-1628.

17. Serio B, Giudice V, Guariglia R, et al. Prophylactic letermovir decreases cytomegalovirus reactivation after stem cell transplantation: a single-center real-world evidence study. *Le infezioni in medicina.* Mar 1 2021;29(1):102-113.

18. Sassine J, Khawaja F, Shigle TL, et al. Refractory and Resistant Cytomegalovirus After Hematopoietic Cell Transplant in the Letermovir Primary Prophylaxis Era. *Clinical infectious diseases : an official publication of the Infectious Diseases Society of America.* Oct 20 2021;73(8):1346-1354.

19. Hiraishi I, Ueno R, Watanabe A, Maekawa S. Safety and Effectiveness of Letermovir in Allogenic Hematopoietic Stem Cell Transplantation Recipients: Interim Report of Post-marketing Surveillance in Japan. *Clinical drug investigation.* Dec 2021;41(12):1075-1086.

20. Beauvais D, Robin C, Thiebaut A, et al. Effective Letermovir Prophylaxis of CMV infection post allogeneic hematopoietic cell transplantation: Results from the French temporary authorization of use compassionate program. *Journal of clinical virology : the official publication of the Pan American Society for Clinical Virology.* Mar 2022;148:105106.

21. Politikos I, Lau C, Devlin SM, et al. Extended-duration letermovir prophylaxis for cytomegalovirus infection after cord blood transplantation in adults. *Blood advances.* Dec 27 2022;6(24):6291-6300.

22. Gabanti E, Borsani O, Colombo AA, et al. Human Cytomegalovirus-Specific T-Cell Reconstitution and Late-Onset Cytomegalovirus Infection in Hematopoietic Stem Cell Transplantation Recipients following Letermovir Prophylaxis. *Transplantation and cellular therapy.* Apr 2022;28(4):211.e211-211.e219.

23. Daukshus NP, Cirincione A, Siver M, et al. Letermovir for Cytomegalovirus Prevention in Adolescent Patients Following Hematopoietic Cell Transplantation. *Journal of the Pediatric Infectious Diseases Society.* Jul 21 2022;11(7):337-340.

24. Richert-Przygonska M, Jaremek K, Debski R, et al. Letermovir Prophylaxis for Cytomegalovirus Infection in Children After Hematopoietic Cell Transplantation. *Anticancer research.* Jul 2022;42(7):3607-3612.

25. Cheng CN, Li SS, Yeh YH, Shen CF, Chen JS. Letermovir prophylaxis for cytomegalovirus reactivation in children who underwent hematopoietic stem cell transplantation: A single-institute experience in Taiwan. *Journal of microbiology, immunology, and infection = Wei mian yu gan ran za zhi.* Apr 2022;55(2):323-327.

26. Freyer CW, Carulli A, Gier S, et al. Letermovir vs. high-dose valacyclovir for cytomegalovirus prophylaxis following haploidentical or mismatched unrelated donor allogeneic hematopoietic cell transplantation receiving post-transplant cyclophosphamide. *Leukemia & lymphoma.* Aug 2022;63(8):1925-1933.

27. Yoshimura H, Satake A, Ishii Y, et al. Real-world efficacy of letermovir prophylaxis for cytomegalovirus infection after allogeneic hematopoietic stem cell transplantation: A single-center retrospective analysis. *Journal of infection and chemotherapy : official journal of the Japan Society of Chemotherapy.* Sep 2022;28(9):1317-1323.

28. Mizuno K, Sakurai M, Kato J, et al. Risk factor analysis for cytomegalovirus reactivation under prophylaxis with letermovir after allogeneic hematopoietic stem cell transplantation. *Transplant infectious disease : an official journal of the Transplantation Society.* Dec 2022;24(6):e13904.

29. Sourisseau M, Faure E, Béhal H, et al. The promising efficacy of a risk-based letermovir use strategy in CMV-positive allogeneic hematopoietic cell recipients. *Blood advances.* Mar 14 2023;7(5):856-865.

30. Giménez E, Guerreiro M, Torres I, et al. Features of cytomegalovirus DNAemia and virus-specific T-cell responses in allogeneic hematopoietic stem-cell transplant recipients during prophylaxis with letermovir. *Transplant infectious disease : an official journal of the Transplantation Society.* Apr 2023;25(2):e14021.

31. Dwabe S, Hsiao M, Ali A, et al. Real world experience: Examining outcomes using letermovir for CMV prophylaxis in high-risk allogeneic hematopoietic stem cell patients in the setting of using T-cell depletion as GVHD prophylaxis. *Transplant immunology.* Feb 2023;76:101769.

32. Zavaglio F, Vitello D, Bergami F, et al. Human Cytomegalovirus (HCMV) - specific T-cell response after letermovir prophylaxis is predictive for subsequent HCMV reactivation in haematopoietic stem cell transplant recipients. *Journal of clinical virology : the official publication of the Pan American Society for Clinical Virology.* Aug 2023;165:105519.

33. Włodarczyk M, Wieczorkiewicz-Kabut A, Białas K, et al. Real-Life Data on the Efficacy and Safety of Letermovir for Primary Prophylaxis of Cytomegalovirus in Allogeneic Hematopoietic Stem Cell Recipients: A Single-Center Analysis. *Turkish journal of haematology : official journal of Turkish Society of Haematology.* Mar 1 2024;41(1):9-15.

34. Febres-Aldana A, Khawaja F, Morado-Aramburo O, et al. Mortality in recipients of allogeneic haematopoietic cell transplantation in the era of cytomegalovirus primary prophylaxis: a single-centre retrospective experience. *Clinical microbiology and infection : the official publication of the European Society of Clinical Microbiology and Infectious Diseases.* Jun 2024;30(6):803-809.

35. McGuirk M, Shahzad M, Amin MK, et al. Predictors of cytomegalovirus reactivation after allogeneic hematopoietic cell transplantation: Insights from a real-world experience. *Transplant immunology.* Jun 2024;84:102039.

36. Jacobs SE, Ibrahim U, Vega AB, et al. Dynamics of cytomegalovirus-specific T-cell recovery in allogeneic hematopoietic cell transplant recipients using a commercially available flow cytometry assay: A pilot study. *Transplant infectious disease : an official journal of the Transplantation Society.* Jun 2024;26(3):e14290.

37. Mendoza MA, Bhaimia E, Alkhateeb HB, Razonable RR, Thoendel M. Which allogeneic hematopoietic cell transplant recipients have an increased risk for delayed-onset clinically significant cytomegalovirus infection after letermovir prophylaxis? *Transplant infectious disease : an official journal of the Transplantation Society.* Dec 2024;26(6):e14377.

38. Tan CA, Palen L, Su Y, et al. Impact of Primary Letermovir Prophylaxis Versus Preemptive Antiviral Therapy for Cytomegalovirus on Economic and Clinical Outcomes after Hematopoietic Cell Transplantation. *Transplantation and cellular therapy.* Aug 2024;30(8):792.e791-792.e712.

39. Russo D, Schmitt M, Pilorge S, et al. Efficacy and safety of extended duration letermovir prophylaxis in recipients of haematopoietic stem-cell transplantation at risk of cytomegalovirus infection: a multicentre, randomised, double-blind, placebo-controlled, phase 3 trial. *The Lancet. Haematology.* Feb 2024;11(2):e127-e135.

40. Han G, Stern A, Lee YJ, et al. Letermovir for Prevention of Recurrent Cytomegalovirus in High-Risk Allogeneic Hematopoietic Cell Transplantation Recipients. *Transplantation and cellular therapy.* Feb 2025;31(2):105.e101-105.e109.
